# Estimation of vaccination coverage from electronic healthcare records; methods performance evaluation – A contribution of the ADVANCE-project

**DOI:** 10.1371/journal.pone.0222296

**Published:** 2019-09-18

**Authors:** Toon Braeye, Vincent Bauchau, Miriam Sturkenboom, Hanne-Dorthe Emborg, Ana Llorente García, Consuelo Huerta, Elisa Martin Merino, Kaatje Bollaerts

**Affiliations:** 1 Sciensano, Brussels, Belgium; 2 Hasselt University, Hasselt, Belgium; 3 GSK Vaccines, Wavre, Belgium; 4 P95 Epidemiology and Pharmacovigilance, Leuven, Belgium; 5 VACCINE.GRID foundation, Basel, Switzerland; 6 University Medical Center Utrecht, Julius Global Health, Utrecht, the Netherlands; 7 Statens Serum Institut, Copenhagen, Denmark; 8 BIFAP database, Spanish Agency of Medicines and Medical Devices, Madrid, Spain; University of Campania, ITALY

## Abstract

**Introduction:**

The Accelerated Development of VAccine beNefit-risk Collaboration in Europe (ADVANCE) is a public private collaboration aiming to develop and test a system for rapid benefit-risk (B/R) monitoring of vaccines, using existing electronic healthcare record (eHR) databases in Europe.

Part of the data in such sources is missing due to incomplete follow-up hampering the accurate estimation of vaccination coverage. We compared different methods for coverage estimation from eHR databases; naïve period prevalence, complete case period prevalence, period prevalence adjusted for follow-up time, Kaplan-Meier (KM) analysis and (adjusted) inverse probability weighing (IPW).

**Methods:**

We created simulation scenarios with different proportions of completeness of follow-up. Both completeness independent and dependent from vaccination date and status were considered. The root mean squared error (RMSE) and relative difference between the estimated and true coverage were used to assess the performance of the different methods for each of the scenarios. We included data examples on the vaccination coverage of human papilloma virus and pertussis component containing vaccines from the Spanish BIFAP database.

**Results:**

Under completeness independent from vaccination date or status, several methods provided estimates with bias close to zero. However, when dependence between completeness of follow-up and vaccination date or status was present, all methods generated biased estimates. The IPW/CDF methods were generally the least biased. Preference for a specific method should be based on the type of censoring and type of dependence between completeness of follow-up and vaccination. Additional insights into these aspects, might be gained by applying several methods.

## Introduction

The evaluation of the performance of vaccination programs requires tools to monitor compliance with the recommended vaccination schedules. This compliance is often summarized using vaccination coverage estimates. The methods and data sources used for coverage estimation vary widely between studies. The need for harmonization is generally acknowledged [1]. Coverage estimates are either reported as a point estimate of the coverage attained at a specific age or date or as multiple age-specific estimates. The advantage of reporting age-specific estimates is that the age-appropriate uptake of vaccination can be assessed. Single point estimates do not reflect the variation in vaccine administration with respect to the recommended age [2]. In the presence of vaccination delay, they misrepresent the vaccine induced protection and herd immunity [3]. The disadvantage of age-specific estimates is that information on the exact age at vaccination is required whereas for single point estimates it is sufficient to know if a person was vaccinated by a certain time or age. Therefore, not all data sources will allow for age-specific coverage estimation.

A survey from 2016 among European countries demonstrated that multiple countries were developing an immunization information system. In this system individual level information on vaccines received in a given area is used to inform both government and individuals [4]. In the absence of an exhaustive immunization registry, surveys (e.g. household surveys) and administrative data (e.g. school health examination reports) are the most typically used data sources [5]. Both have limitations. Household surveys rely on vaccination cards and/or recall. They have been described as both over- and underestimating vaccine coverage due to recall bias and incomplete records [6,7]. For some administrative data sources, such as reimbursement or insurance data, a clear denominator, the number of persons eligible for vaccination, is lacking [8,9]. In an effort to overcome such limitations, data sources have been merged. Administrative data has been combined with immunization registries in the PRISM program [10].

In this paper, we focused on electronic healthcare records (eHRs) as data source. EHRs have been used previously to estimate vaccination coverage [10,11]. They allow for the timely monitoring of age-specific coverage estimates at a relatively low cost and often cover large geographical areas or sizeable populations. Their popularity is growing. The populations captured in eHRs however are generally dynamic, with members moving in and out of the population over time (i.e. transient membership). This results in incomplete follow-up, hampering a straightforward estimation of vaccination coverage from eHRs [12]. As vaccination possibly occurs outside follow-up, coverage estimation not accounting for incomplete follow-up will underestimate vaccination coverage. If we assume no exposure misclassification and independence of completeness of follow-up and vaccination date/status, we can consider the estimation of vaccination coverage from eHRs as a missing completely at random problem [13]. Different statistical methods with good finite sample properties, such as inverse probability weighting (*IPW*), have been developed for such and less restrictive mechanisms of missingness [14]. Of these methods, the Kaplan-Meier (*KM*) method has received most attention in the field of age-specific vaccination coverage estimation [15].

With the Accelerated Development of VAccine beNefit-risk Collaboration in Europe project (ADVANCE) we aim to build a system that can generate information on vaccine coverage, benefits and risks using available European eHR databases. In this work, we investigated the performance of complete case-analysis, *KM* and *IPW* -methods for the estimation of vaccination coverage from eHR databases with a simulation study. We illustrated the methodology by estimating the coverage of Human Papilloma Virus (HPV) and acellular pertussis component containing (aPE) vaccines from the Spanish ‘Base de Datos Para la Investigación Farmacoepidemiológica en Atención Primaria’ (BIFAP) database.

## Methods

### Notation

The time scale we use is the age of a person (in weeks). For easy notation, we introduce the following definitions:

$$A_{i}=in\;follow-up\;(FU)\;during\;age\;i,\;vaccination\;recorded\;at\;age\;i$$

$$B_{i}=in\;FU\;during\;age\;i,vaccination\;recorded\;before\;age\;i$$

$$C_{i}=in\;FU\;during\;age\;i,no\;recorded\;vaccination\;before\;age\;i$$

$$D_{i}=Not\;in\;FU\;during\;age\;i,vaccination\;recorded\;before\;age\;i$$

$$E_{i}=Not\;in\;FU\;during\;age\;i,no\;recorded\;vaccination\;before\;age\;i$$

The total number of persons (*N*) in the population of interest eligible for vaccination is assumed to be constant over age. The population of interest will often be a specific birth cohort.

N=A+B+C+D+E

The proportion of persons in follow-up is age-dependent:

$${FU}_{i,proportion}=\frac{A_{i}+B_{i}+C_{i}}{N}$$

### Estimators

#### Period prevalence (*PP*)

The period prevalence estimate for age *i* is the proportion of vaccinated persons over the total number of persons eligible for vaccination. In other words; *PP*_*i*_ represents the cumulative incidence up to age *i* over all eligible persons in the cohort.

$${PP}_{i}=\frac{A_{i}+B_{i}+D_{i}}{N}$$

#### Period prevalence: Complete case (*PP*_*CC*_)

The *PP*_*CC*_ analysis is performed on a subset of the data containing only persons with a complete follow-up period, as indicated with the subscript. A complete follow-up is defined as follow-up from the start of the vaccination eligible age till the end of the vaccination eligible age. This period will be vaccine specific and is defined by the researcher.

$${PP}_{CC,i}=\frac{A_{cc,i}+B_{cc,i}}{A_{cc,i}+B_{cc,i}+C_{cc,i}}=\frac{A_{cc,i}+B_{cc,i}}{N_{cc}}$$

#### Period prevalence: Follow-up (*PP*_*FU*_)

The *PP*_*FU*_ estimate for week *i* is the number of vaccinated persons in follow-up divided by the number of persons in follow-up during week *i*.

$${PP}_{FU,i}=\frac{A_{i}+B_{i}}{A_{i}+{B_{i}+C}_{i}}$$

#### Kaplan-Meier (*KM*)

We first compute the survival function at age *i* as;

$$S_{i}={\left(\frac{C}{A+C}\right)}_{1}*{\left(\frac{C}{A+C}\right)}_{2}*\ldots *{\left(\frac{C}{A+C}\right)}_{i}$$

The Kaplan-Meier estimate is then defined as one minus the survival function

$${KM}_{i}=1–S_{i}$$

#### Inverse Probability Weighting (*IPW*)

We first compute the proportion of persons in follow-up at age *i*;

$${FU}_{proportion,i}=\frac{A_{i}+B_{i}+C_{i}}{N}$$

The total number of persons vaccinated at age *i* is obtained by weighing the number of recorded vaccinations at age *i* over the proportion of persons in follow-up at age *i*.

$$A_{IPW,i}=\frac{A_{i}}{{FU}_{proportion,i}}$$

The cumulative sum at the end of week *i* of *A*_*IPW*_ is then set as the number of vaccinated persons at age *i*. The *IPW* -coverage estimate is defined as

$${IPW}_{i}=\frac{\sum_{0\to i}A_{IPW,i}}{N}$$

#### Adjusted inverse probability weighting: Cumulative distribution function (*CDF*)

We estimate the cumulative probability density (Φ_*A*_) for the age at vaccination from the subset of persons with a complete follow-up. The cumulative distribution function represents the probability to be vaccinated by a certain age. We interpreted the increase between week *i*−1 (= Φ_*A*_(*t*_*i*−1_)) and *i* (= Φ_*A*_(*t*_*i*_)) as the amount of meaningful follow-up (*MFU*_*i*_). *MFU*_*i*_ thus equals the probability of vaccination during week *i* inferred from persons with a complete follow-up. We use a 5000-step numerical integration to quantify *MFU* for each age-week. Φ_*A*_(*t*_*i*_) represents the total amount of meaningful follow-up at the end of week *i*, Φ_*A*_(*t*_*i*−1_) represents this value at the start of week *i*.

$${MFU}_{i}=\Phi_{A}(t_{i})-\Phi_{A}(t_{i-1}),$$

We subsequently multiply the meaningful follow-up for week *i* with the proportion of persons in follow-up at week *i* to obtain the proportion of meaningful follow-up (*MFU*_*proportion*,*i*_).

$${MFU}_{proportion,i}={FU}_{proportion,i}*{MFU}_{i}$$

To allow for age-specific vaccination coverage estimation we need to normalize the proportion of meaningful follow-up at the end of week *i*;

$${MFU}_{proportion.normalized,i}=\frac{\sum_{o\to i}{MFU}_{proportion,i}}{\sum_{o\to i}{MFU}_{i}}$$

Finally we weight the total number of vaccinations at the end of week *i* by the normalized *MFU*_*proportion*,*i*_.

$${CDF}_{i}=\frac{\sum_{0\to i}A_{i}}{{MFU}_{proportion.normalized,i}}$$

### Software

All formulas were written in R version 3.5.2 and all R-code is made available as supplementary material (S1 R-Code Estimation functions, S2 R-Code Simulation scenarios). We opted for Kernel density estimation as this is a non-parametric method for which no distributional assumptions are needed and used the default R density function (‘density()’) [16].

### Simulation study

Per simulation run we created 10 000 persons. The date of birth of all persons is set at 1^st^ of January 2000. By default, all persons had a follow-up from birth (start-date: 01/01/2000) till one year of age (end-date: 31/12/2001). The vaccination coverage was set at 90% and the age at vaccination was sampled from a Weibull distribution (shape = 1, scale = 30, location = 28). A Weibull distribution is an appropriate distribution for age at vaccination as its longer right tail reflects delayed vaccinations [17]. For the *PP*_*CC*_- and *CDF* -method, complete cases were defined as persons with follow-up from date of birth, 1^st^ of January 2000, till the end of the study period, 31^st^ of December 2000.

#### Simulation scenarios

**Fig 1:**
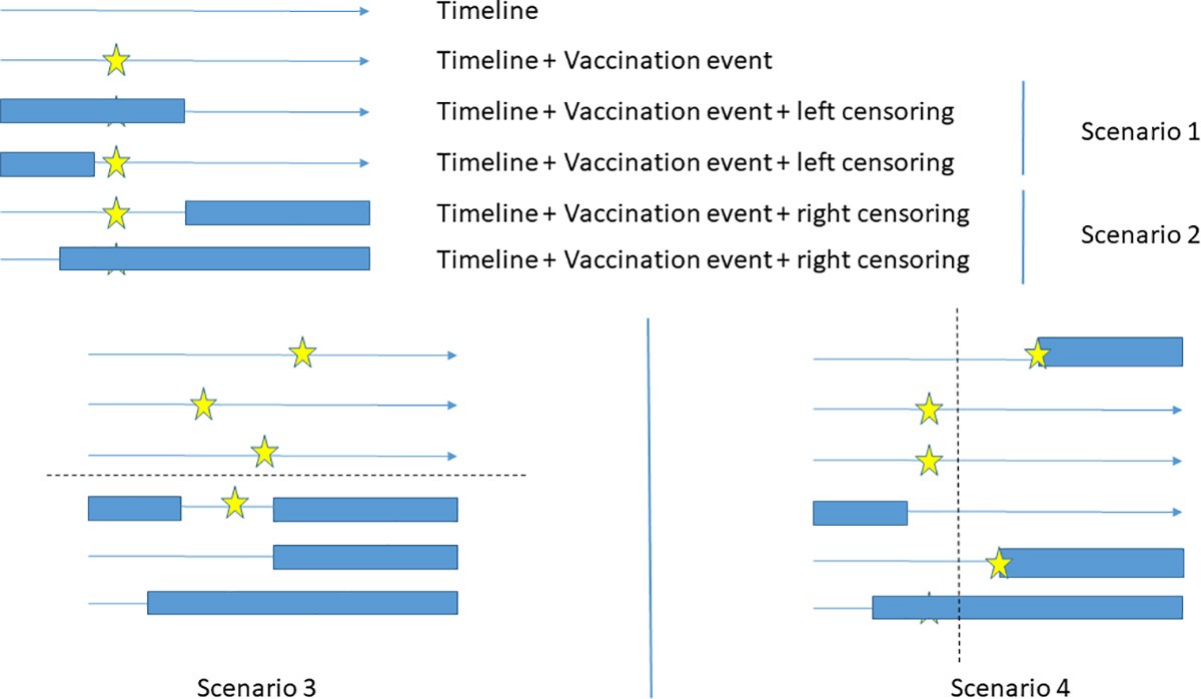
Graphical overview of the simulation scenarios.

Incomplete follow-up was created in the simulated scenarios by altering start-dates (left censoring) and end-dates (right censoring) of follow-up. The proportion of altered start-dates and/or end-dates were 0, 0.3, 0.5, 0.7 and 0.9. We varied the amount of incompleteness in each simulation scenario and defined four scenarios by the type of incompleteness: ‘random left-censoring’ (scenario 1), ‘random right-censoring’ (scenario 2), ‘double censoring dependent on vaccination status’ (scenario 3) and ‘double censoring dependent on age at vaccination’ (scenario 4) (Table 1, Fig 1). We did not allow for re-entry into follow-up. This reflected BIFAP input data; persons were only allowed to have a single start- and end-date. Whenever the altered start-date was a later date than the altered end-date, we randomly chose one of the dates to be set back to its original value.

**Table 1:** Overview of the simulation scenarios.

**Completeness of follow-up independent of vaccination**	
	**Scenario 1** (random left-censoring): Altered start of follow-up dates for a proportion of randomly selected subjects.
	**Scenario 2** (random right-censoring): Altered end of follow-up dates for a proportion of randomly selected subjects.
**Completeness of follow-up dependent of vaccination**	
	**Scenario 3** (double censoring dependent on vaccination status): The vaccination coverage of persons with a complete follow-up is 10% higher than the coverage of persons with incomplete follow-up.
	**Scenario 4:** (double censoring dependent on age at vaccination): If late age of vaccination, 50% chance of end-date at date of vaccination.

In scenarios 1 and 2, we randomly altered the start- (scenario 1) or end-date (scenario 2) of a proportion of randomly selected simulated persons. In scenario 3, we created dependence between follow-up and vaccination status by more frequently altering start- and end-dates in unvaccinated than in vaccinated persons. The alterations were such that the coverage among persons with a complete follow-up was 10% higher than the coverage among persons with an incomplete follow-up. This simulation scenario represents a situation in which a complete follow-up is indicative for compliance with vaccination. In scenario 4, we altered both start- and enddates and created dependence between the period of follow-up and the date of vaccination. More precisely, in 50% of persons who were three months or older at the time of vaccination, the vaccination date coincided with the end-date of follow-up. The population vaccination coverage remained at 90% as in all simulation scenarios. This simulation scenario represents a situation in which persons with low compliance to the vaccination schedule are also more likely to drop-out early.

## Comparing the methods and presenting results

Methods performance was assessed using the root mean squared error (RMSE), defined as

$${RMSE}_{i}=\sqrt{\frac{\sum_{1}^{N}{\left({coverage}_{i,n}–{estimated\;coverage}_{i,n}\right)}^{2}}{N}},$$

and the relative difference (RD), defined as

$${RD}_{i}=\frac{\sum_{1}^{N}\left(\frac{{coverage}_{i,n}–{estimated\;coverage}_{i,n}}{{coverage}_{i,n}}\right)}{N},$$

where *n* represent runs of the simulation (*n* = 1:*N* with *N* = 10 000) and *i* represents the age-estimates in weeks (*i* = 1:*I* with *I* = 52). For each scenario we present three graphs. We first present the RMSE and RD over the proportion of missing FU.

$${missing\;FU}_{i}=1-\frac{A_{i}+B_{i}+C_{i}}{N}$$

In the third graph we present the RD by age in weeks for the highest proportion of incompleteness.

## Data illustration: BIFAP database

Real life data from the BIFAP database on HPV (dose 1) and aPE (dose 2) vaccination coverage was used to illustrate the methodology and assess the impact of censoring. Exploratory analysis had shown that the follow-up of the population captured in the BIFAP database was dynamic and therefore allowed for illustrative examples. The BIFAP-database is a primary care database from the National Health System in Spain [18]. The database is multi-regional. It started registration in 2003 and during 2010 a new region was added to the database. A recent article about the recording process in the BIFAP database, its validation and precision reported that 97.9% of the vaccination records were submitted at the date of vaccination by the nurse who administered the vaccine in the primary care practice. Primary care general practitioners and paediatricians also submit vaccination records to the database [19].HPV-immunization has been included in the Spanish national vaccination program from 2007 onwards for all girls aged 11–14 years old [20]. The second dose of pertussis component containing vaccine is recommended at 4 months of age since 1999. Since 2002 whole cell pertussis vaccine (wPE) has been replaced with aPE [21].

For the first dose of HPV vaccine, our population of interest were females born in 1999, living in Spain and registered in the BIFAP database before the age of 16 years. For the second dose of aPE vaccine, our population of interest were all children born in 2010, living in Spain and registered in the BIFAP database before the age of 30 days. The methods as described for the simulation study were applied. We presented age-specific estimates (by age in years) for the age groups 0–6 years (aPE) and 10–16 years (HPV). While an exact comparison is not possible, because the population and methodology differs, we do also present the estimates of the Spanish public health authority for both HPV and aPE. This estimate is based on the number of vaccines bought by the public healthcare administration over population size.

## Results

### Simulation scenarios 1 and 2

**Fig 2:**
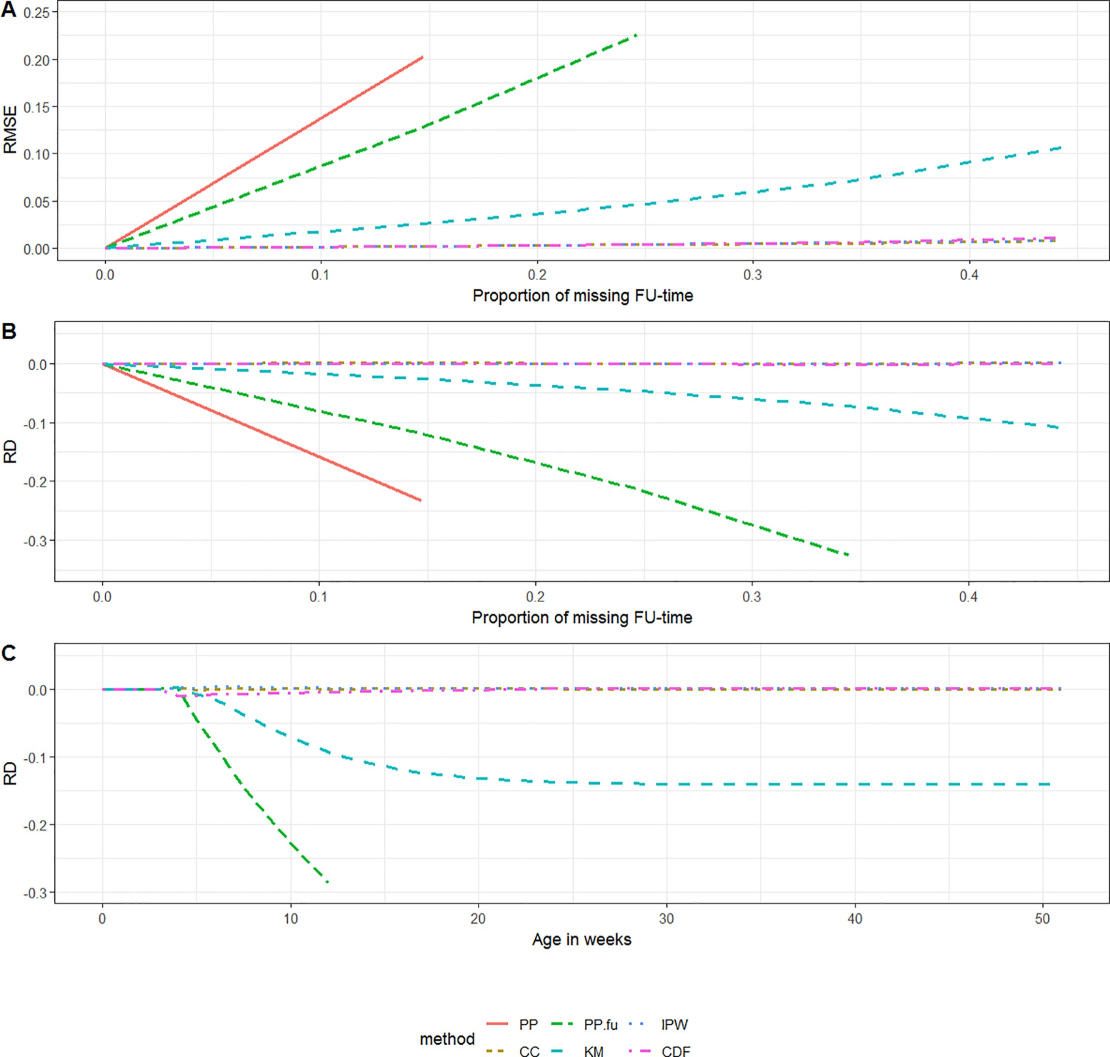
Scenario 1 (random left-censoring). (A) RMSE by the proportion of incomplete follow-up time, (B) RD by the proportion of incomplete follow-up-time and (C) RD by ‘age in weeks’ (***PP*** out of boundaries) *(KM = Kaplan-Meier*, *PP = Period Prevalence*, *PP*.*fu = PP*.*follow-up*, *CDF = Cumulative Distribution Function*, *IPW = Inverse Probability Weighting*, *CC = Complete Case*, *RD = relative difference*, *FU = follow-up*, *RMSE = Root Mean Squared Error)*.

**Fig 3:**
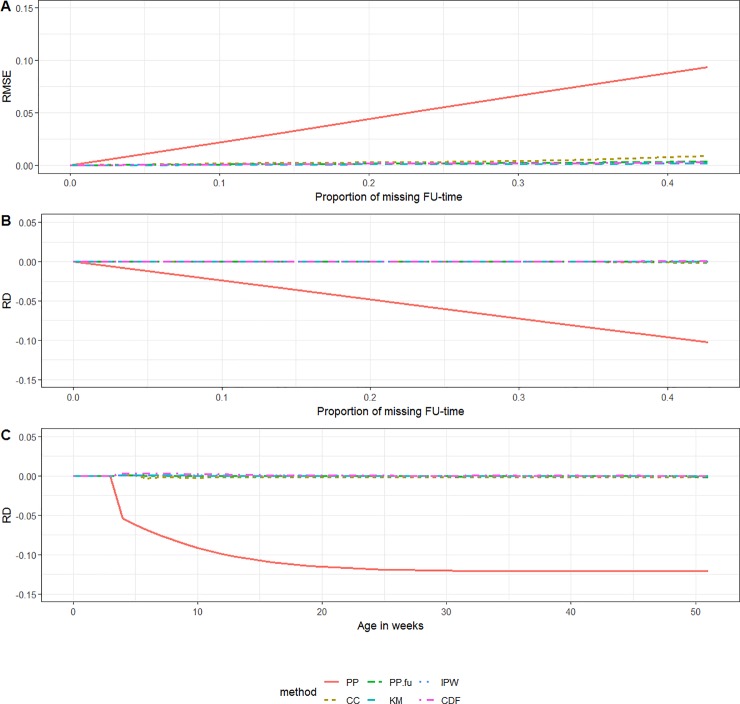
Scenario 2 (random right-censoring). (A) RMSE by the proportion of missing follow-up time, (B) RD by the proportion of missing follow-up-time and (C) RD by ‘age in weeks’ *(KM = Kaplan-Meier*, *PP = Period Prevalence*, *PP*.*fu = PP*.*follow-up*, *CDF = Cumulative Distribution Function*, *IPW = Inverse Probability Weighting*, *CC = Complete Case*, *RD = relative difference*, *FU = follow-up*, *RMSE = Root Mean Squared Error)*.

The RMSE associated with the *PP*-method increased with increasing proportion of missing data. Since vaccinations were more likely registered in the beginning of the follow-up period than towards the end, the RMSE over the proportion of missing data was smaller in scenario 2 than in scenario 1. The performance of the *KM*- and *PP*_*FU*_-method also differed between scenarios 1 and 2. Both methods showed a small to non-existent bias in scenario 2, while they showed substantial bias, increasing underestimation with increasing proportion of missing data, in scenario 1. The *PP*_*CC*_-, *IPW*- and *CDF*-method allowed for close to unbiased estimates in both scenarios 1 and 2, even when a large proportion of subjects was having incomplete follow-up (Figs 2 and 3).

### Simulation scenarios 3 and 4

Scenarios 3 and 4 resulted in biased estimates for all methods. The bias was largest for the *PP*-estimate, followed by the *PP*_*FU*_-estimate and the *KM*-estimate in both scenarios 3 and 4. The performance of the *PP*_*CC*_-, the *IPW*- and *CDF*-method differed between scenarios.

**Fig 4:**
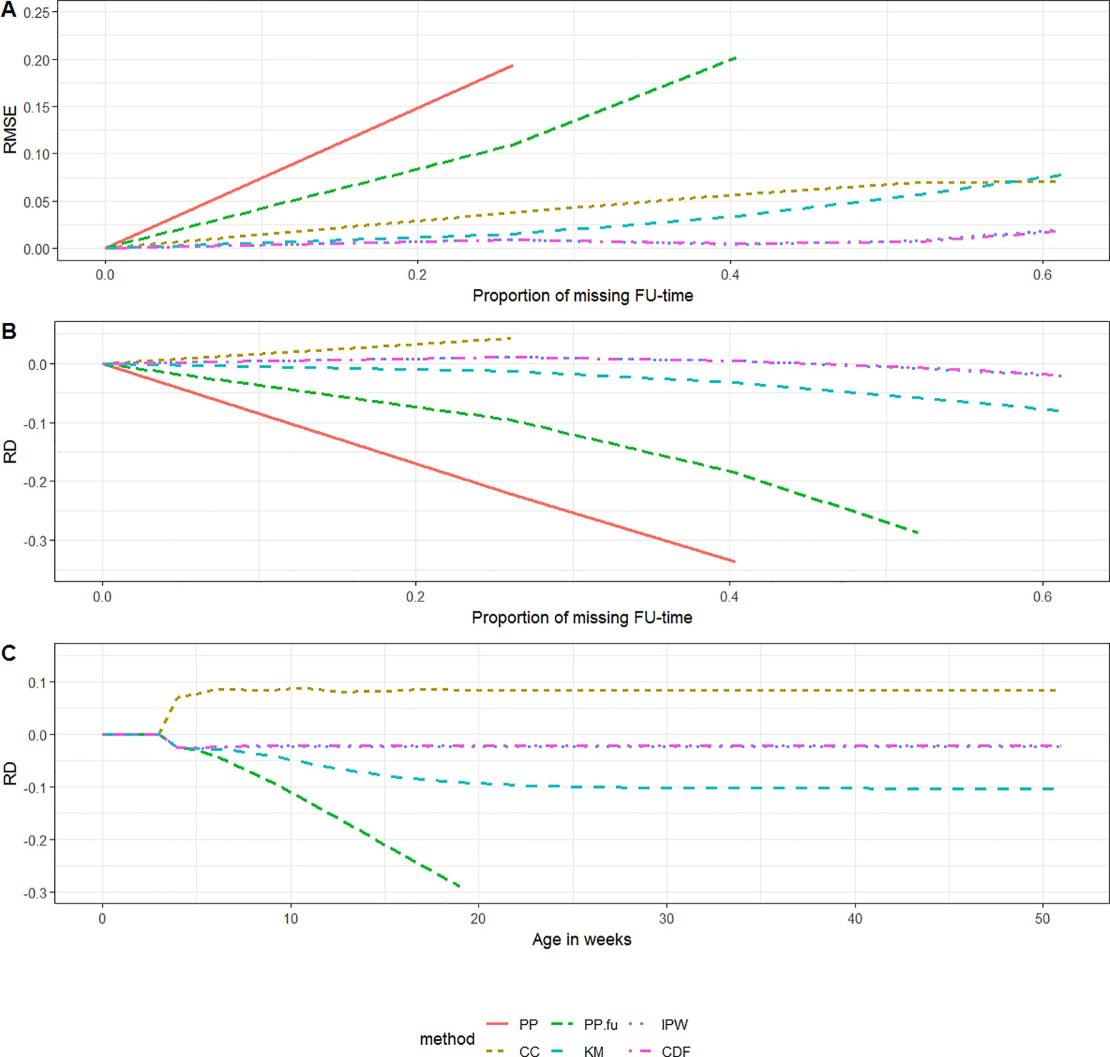
Scenario 3 (double censoring dependent on vaccination status). (A) RMSE by the proportion of missing follow-up time, (B) RD by the proportion of missing follow-up-time and (C) RD by ‘age in weeks’ (***PP*** out of boundaries) *(KM = Kaplan-Meier*, *PP = Period Prevalence*, *PP*.*fu = PP*.*follow-up*, *CDF = Cumulative Distribution Function*, *IPW = Inverse Probability Weighting*, *CC = Complete Case*, *RD = relative difference*, *FU = follow-up*, *RMSE = Root Mean Squared Error)*.

**Fig 5:**
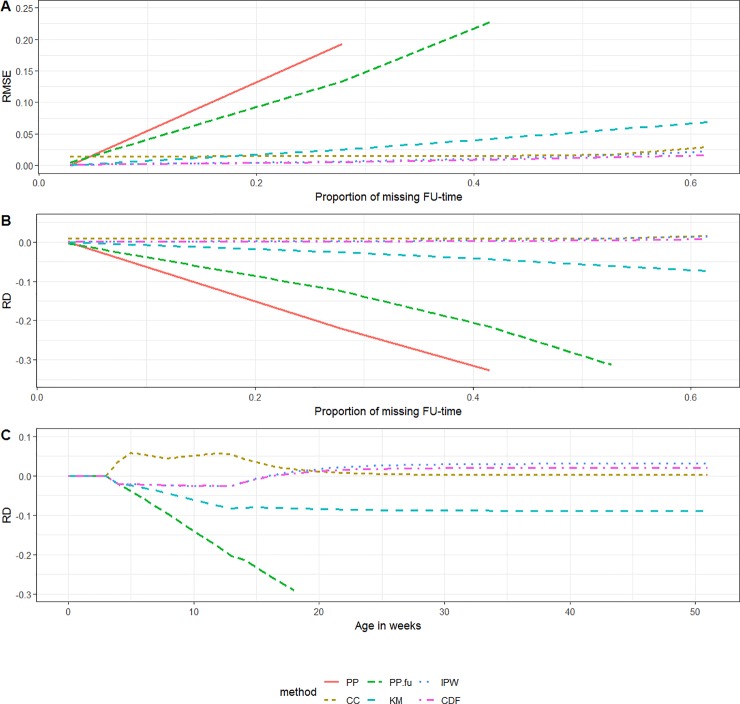
Scenario 4 (double censoring dependent on age at vaccination). (A) RMSE by the proportion of missing follow-up time, (B) RD by the proportion of missing follow-up-time and (C) RD by ‘age in weeks’ (***PP*** out of boundaries) *(KM = Kaplan-Meier*, *PP = Period Prevalence*, *PP*.*fu = PP*.*follow-up*, *CDF = Cumulative Distribution Function*, *IPW = Inverse Probability Weighting*, *CC = Complete Case*, *RD = relative difference*, *FU = follow-up*, *RMSE = Root Mean Squared Error)*.

The *PP*_*CC*_-method showed biased estimation during the age period in which the coverage changed most, from age 3 weeks to 20 weeks. In scenario 3, the *PP*_*CC*_-estimate continued to be more biased than the *IPW*- and *CDF*-estimate. Due to the specific simulation setting (complete cases had a coverage that was 10% higher than persons with incomplete follow-up), the bias caused by overestimation was maximum 10%. In scenario 4 from age 20 weeks onwards, the *PP*_*CC*_-method provided the least biased estimate as compared to the other methods. The RMSE associated with the *PP*_*CC*_-method was always larger than the RMSE associated with the *IPW*- and *CDF*-method since RMSE was aggregated over the age in weeks. Both the *IPW*- and *CDF*-method overestimated the vaccination coverage from age 20 weeks onwards in scenario 4. This overestimation was larger for the *IPW*-estimate than for the *CDF*-estimate (Figs 4 and 5).

### Data examples

#### HPV vaccination

**Fig 6:**
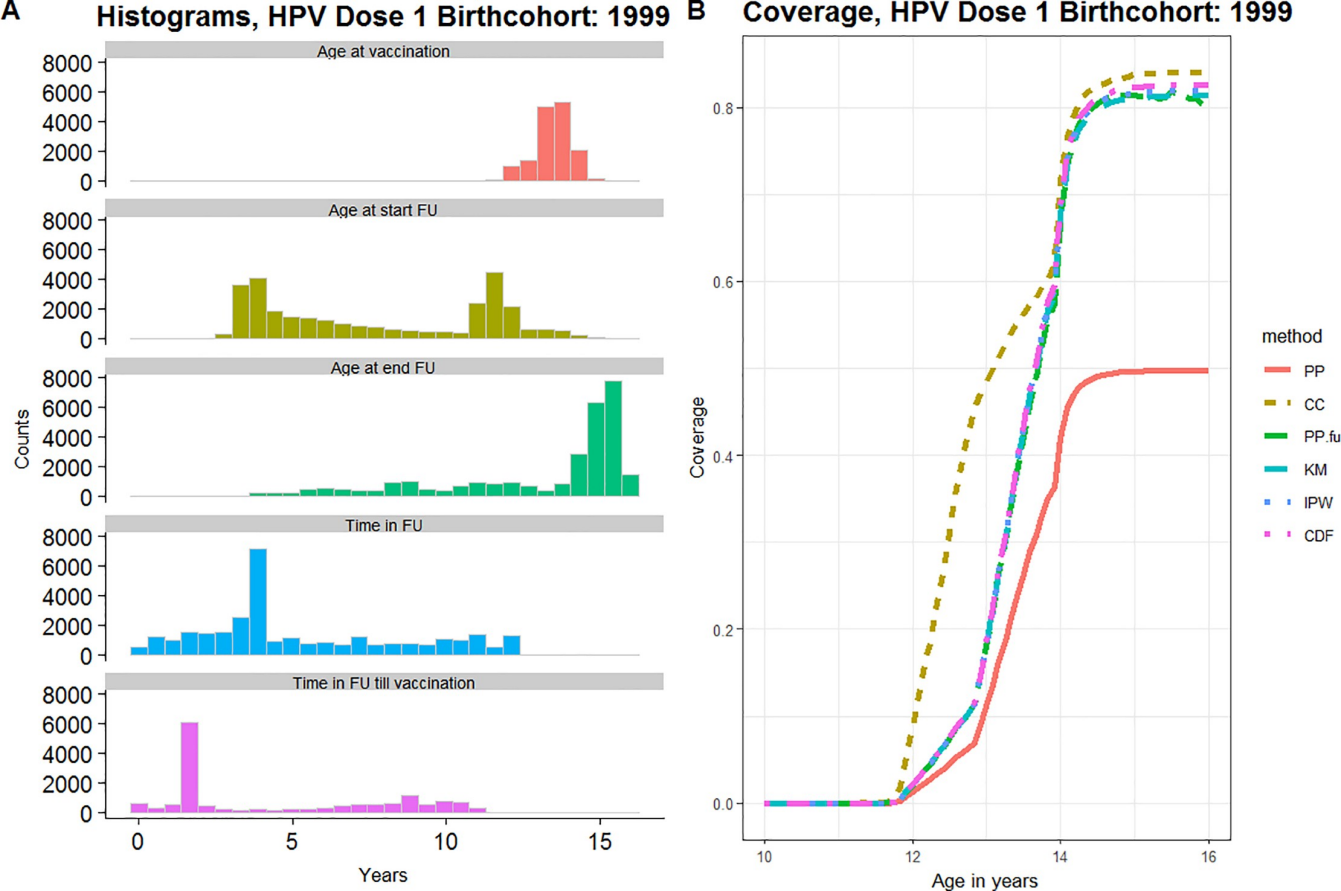
HPV vaccination. (A) Overview of the cohort characteristics (FU = follow-up) presented as counts over years. (B) Age-specific estimates of the coverage over age in years. Both for the first dose of HPV-vaccine, birthcohort of females born in 1999, BIFAP, Spain.

We included 30,170 female persons born in 1999 with at least one day of follow-up in the BIFAP database between 1999 and 2016. For 38% and 65% of the girls, follow-up started after the age of 10 years and ended before the age of 15 years respectively, implying both left and right censoring of the follow-up time (Fig 6, left panel). The median follow-up time was 3.8 years (interquartile range = 4.8 years). Complete cases were defined as having continuous follow-up from 10 to 15 years of age (N = 4266, 14.1%). The date of vaccination and the start of follow-up occurred simultaneously for 382 girls.

Because of censoring, the *PP*-estimate (49.6%) underestimated the vaccination coverage (Fig 6, right panel). Because of left-censoring the *KM*-estimate (81.0%) and the *PP*_*FU*_-estimate (81.3%) at 15 years of age also likely underestimated the coverage. Because of right-censoring the *PP*_*FU*_-estimate became less stable from 15 years of age on. Because there were only 7.1% (N = 4266) complete cases, the *PP*_*CC*_-estimate (83.7%) was considered less representative. We preferred the *CDF*/*IPW* -estimate over the other estimates and estimated the coverage for Spanish women born in 1999 under follow-up by the BIFAP-database at 81.6 (IPW) - 82.3 (CDF) % by the age of 15 years. The Spanish public health authority estimated the first dose HPV-vaccine coverage at 74.0%-91.4% in 2015 for the specific regions that also provide primary care data to BIFAP [22].

#### aPE vaccination

**Fig 7:**
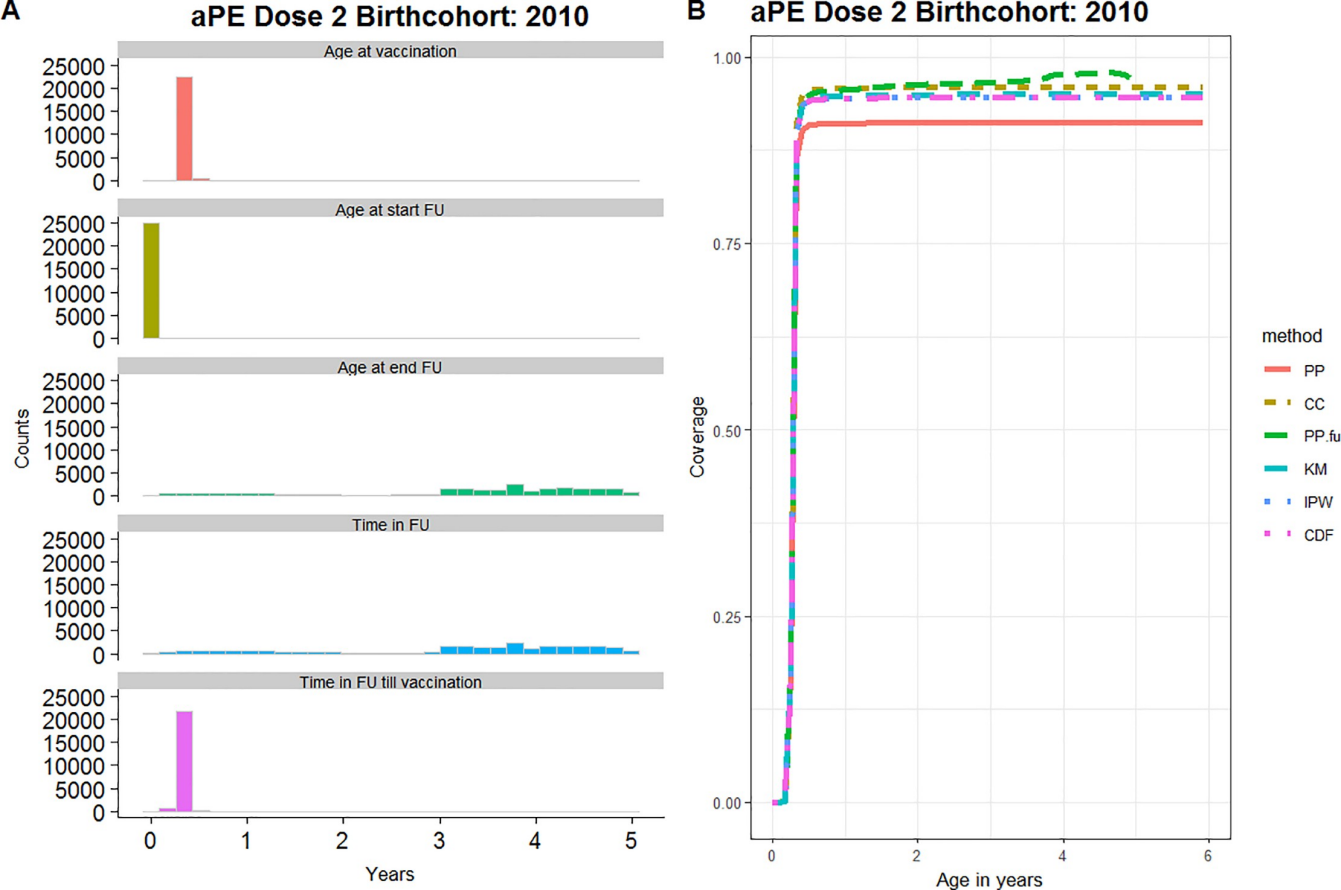
aPE vaccination. (A) Overview of the cohort characteristics (FU = follow-up) presented as counts over years. (B) Age-specific estimates of the coverage over age in years. Both for the second dose of aPE containing vaccine, birthcohort of children born in 2010, BIFAP, Spain.

We included 25,078 children born in 2010 with at least one day of follow-up in the BIFAP database between 2010 and 2017. To reduce left censoring the analysis was restricted to children with follow-up before the age of 30 days, which meant that 80% of all children in the 2010 BIFAP-birth cohort that were under follow-up before the age of 6 years were excluded. The follow-up ended before the age of four years for 64% of included children (Fig 7, left panel). The median duration of follow-up was 3.7 years (the interquartile range 1.4 years). Complete cases were defined as having continuous follow-up from 30 days to 4 years of age (N = 9028).

Because of right censoring during the period in which vaccinations were registered, the *PP*-estimate (91.2% at the age of 4 years) was lower than the other estimates (Fig 7, right panel). The *PP*_*FU*_-estimate (97.7%) indicates right censoring of mostly unvaccinated children, as it increased while other estimates remained stable. The positive relation between follow-up and vaccination status is also seen in the *CC*-estimate (96.0%) as this estimate is higher than the *IPW* (94.6%), *KM* (95%) and *CDF* (94.6%)-estimates. Because we selected a study population without left censoring, the *KM*-estimate is not below the *IPW* and *CDF*-estimate. For this study population we preferred the *IPW*, *CDF* or *KM*-method with the aPE dose 2 coverage estimated at 94.6 (*IPW*/*CDF*) - 95 (*KM*) % by the age of 4 years for Spanish children born in 2010 whose follow-up by the BIFAP- database started before the age of 30 days. The Spanish public health authority estimated the second dose aPE-vaccine coverage at 94.1% in 2011 (for children aged 1–2 years old) [22].

## Discussion

In this article we explored the performance of different methods for the estimation of vaccination coverage from eHRs through a simulation study. We compared methods that ignore incompleteness in follow-up (*PP*), circumvent it by subsampling (*PP*_*CC*_, *PP*_*FU*_) or adjust for it (*KM*, *IPW*, *CDF*).

Whenever there is independence between vaccination and incompleteness, the *IPW* and *CDF* -method will provide nearly unbiased estimates even when the proportion of missing data is large. Additional criteria will determine the performance of the other methods. *KM* and *PP*_*FU*_-methods might be considered when left-censoring is absent. If the population of complete cases is large enough *PP*_*CC*_-methods can be considered as well. In case of dependence between completeness of follow-up and vaccination, the estimates vary across methods and all methods provide biased estimates, with *CC*, *IPW* and *CDF* being the least biased for our simulation settings.

The period prevalence (*PP*), complete case-analysis (*PP*_*CC*_), and the period prevalence of the persons in follow-up (*PP*_*FU*_) all estimate the coverage as the number of vaccinated persons over the number of persons in follow-up, but do so over a different subset of the data. The *PP*-method is the only method not accounting for incomplete follow-up. The bias of the *PP*-method will always be downwards and the estimates can therefore be used as a lower bound of the vaccination coverage. For the *PP*_*CC*_-method, only complete cases were used for the calculation of the vaccination coverage. The *PP*_*CC*_-estimates will be biased when the sample of complete cases is not representative for the population of interest in terms of vaccination coverage and age at vaccination. The denominator of the *PP*_*FU*_-method varies according to those actually in follow-up during week *i*. *PP*_*FU*_-estimates can become unstable or decline over time. In case of left-censoring, *PP*_*FU*_-estimates will be biased downward even under independence between vaccination and completeness as persons entering the database after vaccination, thus without having the vaccination registered, will be included in the analysis as unvaccinated.

Survival analysis methods, such as the *KM*-method, have been used previously to assess vaccination coverage and delay in age-specific vaccination [15,23]. The methodology also allows for further analysis, such as research into factors associated with delayed administration of the vaccines through Cox regression analysis [24]. The method as presented in this paper does not correct for left censoring. Researchers who want to use the *KM*-method on dynamic eHRs will have to resort to sub-setting their data to persons with only right censoring (as we did in our data example on pertussis vaccine), or left truncate their data to lose the left censoring. The latter will require additional methodology, such as Turnbull’s self-consistent estimators for doubly censored data [25,26]. Our study did not investigate survival-based methods allowing for inference from doubly censored data [27,28].

The weight in the *IPW*-method was only determined by the total proportion of persons in follow-up at a certain age. Additional stratification might be necessary. For example, if we assume that the proportion of persons in follow-up are year of birth specific, then it is necessary to obtain weights for each birth year to be able to estimate ‘birth year’-cohort specific coverage. The *CDF*-method, a special case of inverse probability weighting, exploits the fact that vaccines are often given at specific ages by using weights corresponding to the probability of vaccination at a specific age and the proportion of persons in follow-up at this age. The probability distribution of age at vaccination is estimated from the age at vaccination of complete cases. A wide range of density estimation techniques can be used for the *CDF*-method, either non-parametrically or parametrically [29]. This identifies two weaknesses of the method. Complete cases with a representative age at vaccination need to be identified and the method is computationally more intensive than the other methods as we need to first estimate a density function and then apply a method such as numerical integration.

The *CDF*-method resulted in less correct estimates in scenarios 1 and 2 (without dependence) during the age periods in which most vaccines were administered as compared to the *IPW*-method. However, in scenario 4 (with dependence) the *CDF*-method outperformed the unadjusted *IPW*-method. The adjusted weights came with the advantage that vaccinations administered at an age within the tail of the estimated ‘age at vaccination’ density distribution contributed less to the *CDF*-estimate then to the *IPW*-estimate. The *IPW*-method therefore overestimated the number of unregistered vaccination to a larger extent compared to the *CDF*-method. One might prefer the *PP*_*CC*_-estimate when dependence between the age at vaccination and the age at the start/end-date of follow-up is present. Especially when the dependence manifests at an ‘early’ age as compared to the other ages at vaccination. For example when healthcare is organised in a way in which entry into the database is linked to contact with the vaccinator.

Insight into the type of censoring and type of dependence between completeness and vaccination are important to select the preferred method for estimating vaccination coverage. This insight can be gained from histograms, as with the BIFAP-examples, but it might also prove helpful to plot and compare estimates from different methods. The *PP*-method will, for example, provide a lower bound and differences between the *KM*- and the *IPW*/*CDF*-method will indicate left censoring. If the shape of the curve of age-specific estimate is different between *PP*_*CC*_ and the other methods, it might reflect an unrepresentative population of complete cases.

### Limitations

The choice of stratification granularity defines the balance between computational time and accuracy of the estimates. We opted for weekly stratification because of the computation burden already inherent to a simulation study. The simulated dates of follow-up and vaccination were however defined on a daily level. As a consequence, none of our methods could produce unbiased estimates.

Martin-Merino et al. recently discussed the data quality of the HPV-records in the BIFAP database and found that presence of a vaccination record confirmed vaccination and the date of vaccination [19]. We did not further investigate the influence of data quality problems. A common problem in obtaining age-specific estimates from eHR databases is that dates of birth are sometimes rounded to month-year of year of birth to protect the patient’s privacy.

Relevant background characteristics (ideology, socio-economic status, …) might influence both follow-up by a database and vaccination and introduce bias into the estimation. It is important to differentiate incomplete follow-up from completely missing follow-up. While the differentiation might be artificial in actual applications, only the former is explored in this paper in simulation scenarios 3 and 4. The latter is outside the scope of our current work. As a result, estimates obtained with any of the methods only estimate coverage for the population captured by the database and, given dependence between vaccination and follow-up, we will only be able to estimate that coverage up to a certain extent. Additional analysis or assumptions on the representativeness of the database-population are necessary to translate estimates to a larger population.

## Supporting information

S1 R-Code Coverage estimation. Functions for coverage estimation.
(ZIP)

S2 R-Code Simulation_scenarios. The four simulation scenarios.
(ZIP)
